# The role of social, demographic and territorial factors in the late detection of hip dysplasia in children in the Republic of Kazakhstan

**DOI:** 10.1186/s13052-022-01375-7

**Published:** 2022-11-17

**Authors:** Zhanna Tastanbekova, Roza Karabekova, Vassiliy Lozovoy, Aleksandr Angelov, Zhomart Suleimenov, Rimma Khuzhakhmedova

**Affiliations:** 1 Department of Pediatric Surgery, Astana Medical University, Beybitshilik str., 49A, Astana, Kazakhstan 010000; 2 Department of Pediatric, South Kazakhstan Medical Academy, Al farabi square 1, Shymkent, Kazakhstan 160000

**Keywords:** Children, Hip dysplasia, Congenital hip dislocation, Late diagnosis, Limited access to medical care, Rural areas

## Abstract

**Background:**

The aim of this research is to identify and study the role of social, demographic and territorial factors in the late detection of children with hip dysplasia.

**Methods:**

We conducted a retrospective cohort study of epidemiological data of patients treated in a hospital in the department of orthopedics of the unitary enterprise based on the right of "Multidisciplinary children's municipal hospital No.2" Nur-Sultan (Kazakhstan) in the period from September 2019 to February 2021. The analysis of archival case histories of 309 patients was carried out. There were 214 early and 95 late detections of this disease. Late detection of hip dysplasia was significantly more likely at birth in cranial presentation (81%, *p* <0.004).

**Results:**

Two-dimensional analysis also showed that late detection was more likely in patients from rural areas (228 children, 73.8%, *p* < 0.001), and that (26 children, 11.4%, *p* = 0.005) these were children from regions with lower income (42500 tenge per month, *p*<0.001). There were also significant differences (*p* = 0.015) in the early (214 children, 69.26%) and late (95 children, 30.7%) diagnosis of hip dysplasia among children whose parents used a national cradle with tight swaddling (95% CI: 1.16 – 4.49).

**Conclusions:**

In our study, we found that children from rural regions of the Republic of Kazakhstan, indigenous Kazakh nationality, using the national cradle in their everyday life, as well as from regions with low average incomes, were significantly more likely to be exposed to late detection of hip dysplasia.

## Introduction

According to literature data, the frequency of hip dislocation at birth is 1 case per 1000, and the frequency of hip subluxation or hip dysplasia is 10 cases per 1000 children born [1]. The correct anatomical development of the hip joint implies the presence of a spherical femoral head located in the acetabulum. Previous studies of the medical history of children have shown that subluxation without adequate treatment leads to an early degenerative process in the hip joint, and untreated bilateral hip dislocations can lead to lumbar hyperlordosis and chronic pain in the lumbar region [2]. Early detection and treatment of hip dysplasia play a major role in a healthy, properly functioning hip joint in adulthood, and studies have shown that the earlier age of the child at the time of initial detection and treatment of hip dysplasia directly correlates with better results of the radiological picture of the hip joint during treatment [3].

Risk factors for the occurrence of hip dysplasia are breech presentation in the prenatal period, belonging to the female sex, burdened family history and the fact of the birth of the first child in the family [4]. There is also conflicting evidence that children born by caesarean section may have a lower risk of developing hip dysplasia [5]. Prematurity was also considered a predisposing factor until recently, but recent studies have shown that premature babies have the same risk of developing hip dysplasia as mature babies [6]. It is noted that 88% of the detected hip joint abnormalities are spontaneously cured by the age of 2 months [7], and, on the contrary, a late diagnosis of hip dysplasia can lead to early degenerative arthritis and an increase in indications for hip arthroplasty [8–10]. Detection of hip dysplasia in newborns during the physical examination is classically carried out using the techniques described by Barlow [7] and Ortolani [11]. Previously, a simple X-ray examination of the hip joints was the gold standard for the diagnosis of hip dysplasia, but in the last few decades, the tendency to use ultrasound examination prevails for diagnosis at an early age [12]. The use of Pavlik harness is the first line of treatment for hip dysplasia, which does not pass spontaneously during the transition to surgical open reposition, when conservative treatment is unsuccessful. Avascular necrosis of the femoral head is a recognized complication of treatment [13, 14]. Initial conservative treatment using the Pavlik harness is carried out when conservative treatment is ineffective at an early age, and the earlier hip dysplasia is diagnosed, the lower the risk of avascular necrosis of the femoral head [15]. For example, the incidence of admissions of young patients for developmental hip dysplasia in Italy is 2.33 cases/100,000 inhabitants (from 2001 to 2016). Developmental hip dysplasia requires early diagnosis and treatment; however, no international consensus on screening protocol and treatment is provided in the literature. Epidemiological studies are helpful to understand the national variation of a specific surgical procedure and compare them with other countries. Data provided by different countries could allow researchers to provide international guidelines for developmental hip dysplasia screening and treatment [16]. Therefore, in September 2018, an international meeting of doctors of various disciplines, with expertise in the detection and treatment of hip dysplasia, was held in Csolyospalos, Hungary. The aim was to achieve consensus on the detection and early treatment of the condition and to develop a standardized system of teaching and training for a hip ultrasound [17].

The principles of screening programs in children at risk are aimed at early detection of hip dysplasia and allow predicting a more favorable fate. However, the development of a successful screening program is a difficult task, since only 25% to 30% [18] of patients with hip dysplasia have identified risk factors.

It is noted that in 2016, the American Academy of Pediatrics issued clinical recommendations that recommend considering the possibility of ultrasound imaging in young children with a high risk of developing hip dysplasia (includes a breech presentation in the prenatal period, positive family history, hypertonicity of the lower extremities, tight swaddling) and negative medical examination results [19].

In the Republic of Kazakhstan, the analysis of the role of late detection of hip dysplasia in the context of determining social, demographic and territorial factors has not been carried out. Only in some scientific works and dissertations, the analysis of morbidity in the context of individual regions of Kazakhstan was conducted. According to the data, most of the late-treated patients were observed in South Kazakhstan, Almaty, Kyzylorda and Zhambyl regions, in rural areas [20–22].

**Fig. 1:**
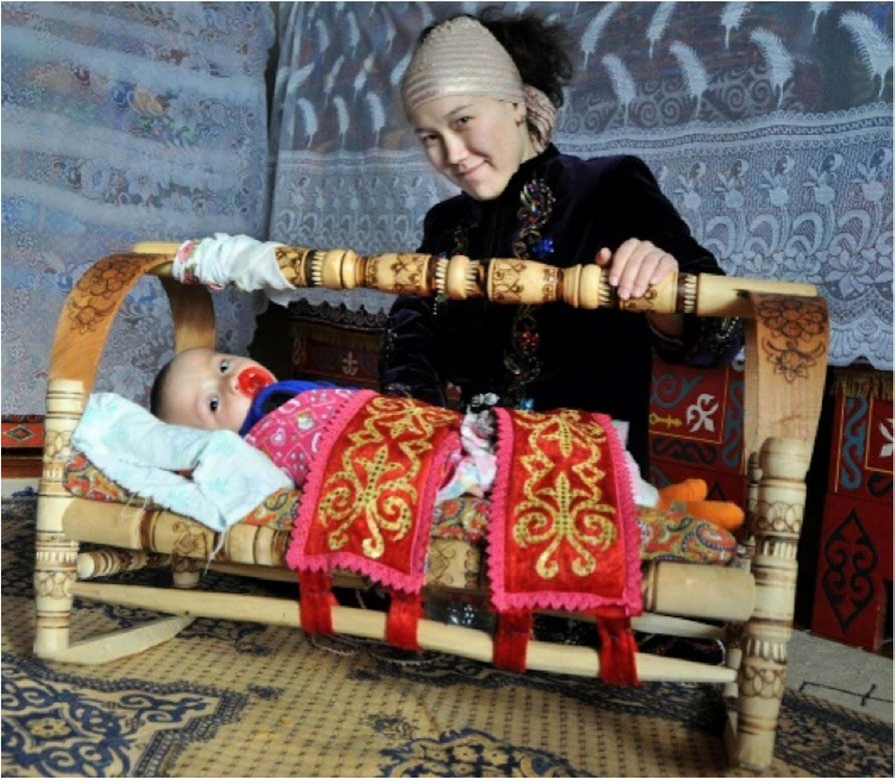
Commonly used «besik»

Despite the hot climate in the south of the country, observing national traditions, residents prefer tight swaddling, and also have a low-income level, where the average monthly salary is according to the data for the second quarter of 2020 - in the Turkestan region (113.3 thousand tenge), while in the city of Nur-Sultan 241.3 thousand tenge, and the minimum wage is 42.5 thousand tenge throughout the Republic [23, 24]. Approximately 80% of children of the indigenous population of Kazakhstan, following national traditions, continue to swaddle newborn babies in homemade wooden cots "besik" in the first few months of life. Kids spend most of their time in this rocking chair with their thighs tightly bound [25, 26]. Figure 1 shows some commonly used "besik".

For example, in the Russian Federation, the incidence of congenital hip dislocation is approximately 5 cases per 1000 newborns, and in ecologically unfavorable regions up to 12% [27]. It is believed that in the Republic of Kazakhstan, the "true" prevalence of hip dysplasia requiring treatment is from 5 to 10 per 1000 newborns [28].

The aim of our current study was to assess the relative proportions of different groups of Kazakhstan is between early and late-arriving patients with hip dysplasia and to assess differences in socio-economic status using indicators of average income in the context of a certain region of the Republic of Kazakhstan. We expected that late-identified patients with hip dysplasia would be people of indigenous nationality (Kazakhs), from rural areas, and from regions of the Republic of Kazakhstan with lower income. As we continue to identify and treat cases of late treatment of hip dysplasia, this study is aimed at a clearer definition of the role of some factors associated with late diagnosis in order to optimize the results of campaigning and diagnostic work.

## Methods

### Patients and methods

On the basis of the unitary enterprise based on the right of "Multidisciplinary children's municipal hospital No.2" in the department of orthopedics of Nur-Sultan, we conducted a retrospective cohort study of hospitalized patients diagnosed with hip dysplasia for the period from September 2019 to February 2021. Patients were identified using ICD-10 codes that were taken from case histories. No formal consent is required for this type of research. The medical records of the selected patients met the criteria for inclusion in the study group, all patients were diagnosed with hip dysplasia and/or hip dislocation, who first applied to our clinic. The criteria for exclusion from the study were nosologies such as hip dysplasia/dislocation associated with other diseases (for example, spinal hernias, cerebral palsy, arthrogryposis, achondrodysplasia of the skeleton, and other teratological causes).

The patients were divided into two groups depending on their age at the initial visit to the clinic. Early treatment of patients with hip dysplasia is considered to be up to six months old, while late treatment is at the age of six months or older. The choice of the age limit of six months was not chosen by chance and is due to the fact that the ineffectiveness of conservative treatment after 6 months of the patient's life is 50% higher [29]. Patient data (address, place of residence, gender, family history, anamnesis of the disease, features in childbirth (cranial/breech presentation), the use of the national cradle "besik") were collected from medical records. According to ethnicity, the patients were divided into 2 groups: Kazakh nationality and non-Kazakh (Russians, Germans, Ukrainians, etc.). The data of the home address and place of residence were also taken from case histories.

All procedures performed in studies involving human participants were in accordance with the ethical standards of the institutional and/or national research committee and with the 1964 Helsinki Declaration and its later amendments or comparable ethical standards. The research was approved by the local ethics committees of Astana Medical University. Informed consent was not required to be obtained as this study was a retrospective review of medical records.

### Statistical analysis

Categorical data was described with absolute values and percentages. The comparison of percentages in the analysis of multipole conjugacy tables was performed using the exact Fisher criterion (with values of the expected phenomenon less than 10). A posteriori comparisons were performed using Pearson's chi-square criterion with Holm correction. The comparison of percentages in the analysis of four-field conjugacy tables was performed using the Pearson chi-square criterion (with values of the expected phenomenon greater than 10). Statistical significance was established at the level of *p* ≤ 0.05. As a quantitative measure of the effect when comparing relative indicators, we used the odds ratio indicator with a 95% confidence interval (95% CI).

## Results

A total of 309 patients (85.8%-265 female, 14.2% -44 male) were included in this study. There were 214 (69.26%) early and 95 (30,7%) late detections, among whom the primary 252 children, 81.6% were Kazakh, 57 children, 18.4% non-Kazakh nationality, according to the total number of patients. According to data analysis, it was found that later detection was more likely in patients from rural areas (228 children. 73.8%, *p*<0.001), as well as data from the place of residence of patients showed that (11.5%, *p* = 0.005) children from regions with lower incomes (42500 tenge per month *p* <0.001). Categorical analysis showed that gender (*p*=0.683), the time of hip dysplasia determination (*p*=1), the type of childbirth (*p*=1) were not statistically significant when compared with the "family history" indicator. However, the number of early diagnosed patients with bilateral form is significantly higher (100, 46,7% vs. 17, 17,9% (*p* < 0.001)="">) than the later definition of this pathology, as well as in unilateral form (left 54, 25,2% vs. 36, 37,9%, right 60, 28% vs. 42, 44,2%), as indicated in Table 1.

**Table 1:** Indicators of early and late detection, depending on the side of the hip joint lesion

Indicator	Fate category/Measurement unit	Detection time		P
		late	early	
	bilateral	17 (17,9%)	100 (46,7%)	< 0.001*="">
Hip dysplasia side,	left side	36 (37,9%)	54 (25,2%)	bilateral - left = 0.006*
abs. (%)				bilateral - right < 0.001*="">
	right side	42 (44,2%)	60 (28%)	

To describe the influence of the use of tight swaddling (putting the baby in the cradle "besik") in the early and late diagnosis of hip dysplasia, we conducted an analysis using the Pearson chi-square criterion technique. In accordance with Table 2 below, when comparing the indicator "use of "besik"" depending on the indicator "detection time", statistically significant differences were revealed (*p* = 0.015). The chances of those who did not use "besik" in the group of early detection were 2 times higher, compared with the group of late diagnosis, the odds differences were statistically significant (95% CI: 1.16 – 4.49).

**Table 2:** Indicators of early and late detection in groups using the national cradle

Indicator	Fate category/Measurement unit	Using the "besik"		p
		Yes	No	
Detection time, abs. (%)	Late	89 (93.7%)	6 (6.3%)	0.015*
	Early	163 (76,1%)	51 (23,8%)	

**Fig. 2:**
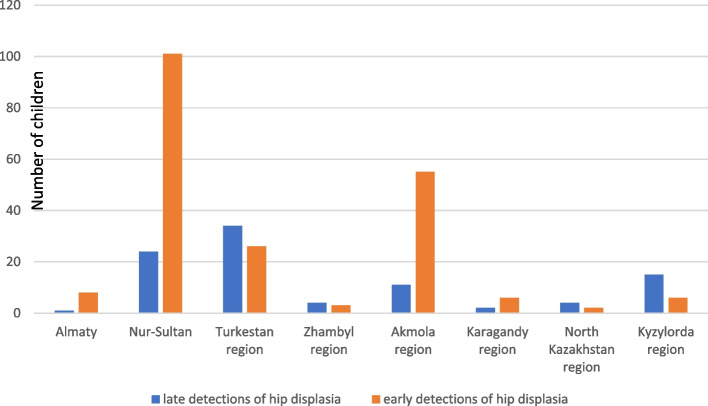
Distribution of children with early and late diagnosis of hip dysplasia by regions of the Republic of Kazakhstan

Over the past 10 years, the number of residents who migrated from the southern regions of the Republic of Kazakhstan to the city of Nur-Sultan has increased several times, but most of them did not have registration at their place of residence. Of the 309 patients registered at the Multidisciplinary City Children's Hospital No. 2, there were residents from different regions of the country. There were cases when patients purposefully came to our clinic for an orthopedic consultation. Below is Fig. 2, which is divided into groups of late and early diagnosis.

According to Fig. 2, the largest number of late detection was observed in patients from the Turkestan region (34 cases). The analysis of the indicator "detection time" was also carried out depending on the indicator "place of arrival". We used the Fisher exact criterion method for multi-field tables, as a result of evaluating the indicator "detection time" depending on the indicator "place of residence", statistically significant differences were established (*p* < 0.001). One of the criteria of the study was to assess the influence of territorial factors and the use of tight swaddling (laying in the Kazakh cradle "besik") on the late diagnosis of hip dysplasia among our patients, for this purpose, an accurate Fischer analysis was conducted, significant differences were revealed (*p* = 0.045), as indicated in Table 3.

**Table 3:** Use of the national cradle by regions of the Republic of Kazakhstan

Indicator	Fate category/Measurement unit	Using the "besik"		p
		yes	no	
	Almaty	8 (3,4%)	1 (1,4%)	
	Nur-Sultan	97 (41,3%)	28 (37,8%)	
	Zhambyl region	5 (2,1%)	2 (2,7%)	
	North Kazakhstan region	5 (2,1%)	1 (1,4%)	
	Akmola region	46 (19,6%)	20 (27%)	
Address, abs. (%)	Karagandy region	2 (0,9%)	6 (8,1%)	
	Kyzylorda region	24 (10,2%)	4 (5,4%)	0.045*
	Turkestan region	48 (20,4%)	12 (16,2%)	

Also, one of the important tasks is to determine the influence of the use of the Kazakh cradle "besik", in which tight swaddling of the lower extremities of the child is used on the late diagnosis of hip dysplasia. Based on the results of our analyses, we can note that 89 children, 93,7% versus 6, 6,3% (*p*=0.015) of cases of late detection of hip dysplasia, tight swaddling of children was used. Despite the fact that current medical recommendations on screening the hips of newborn children and a number of recent studies around the world confirm the existence of a direct link between the increased risk of acquiring hip dysplasia and the tradition of tight swaddling of straightened legs of infants [30–32], in many regions of our country, indigenous people still practice the use of the national cradle "besik", which leads to the above results.

Thus, in our study we used publicly available territorial census data on minimum and average income based on the national population census of the Republic of Kazakhstan and our analysis of data from case histories on the impact of social and territorial factors in the late diagnosis of hip dysplasia is presumptive (our results are based only on the number of patients examined by us at the Municipal Children's Hospital No. 2 of Nur-Sultan), the results of our work imply that these factors are relevant and require further detailed study.

## Discussion

The current clinical recommendations of the American Academy of Pediatrics contain guidelines for screening the hip joints of all newborns with a physical examination and an algorithm for re-examination, referral to a specialist orthopedist and X-ray examination [33]. According to the latest data, the American Academy of Orthopedic Surgeons recommends X-ray examinations only for those six-month-old children who are at risk (breech presentation at birth, burdened family history or clinical instability in the medical history). They also noted the lack of convincing evidence to support universal ultrasound screening of newborns [34, 35]. In other countries, especially in Europe and Australia, universal ultrasound screening is being conducted to try to cover all cases of hip dysplasia at the earliest age, but the debate between universal and selective ultrasound screening is still ongoing [36, 37]. According to a recent Cochrane review, neither ultrasound strategy improved further clinical outcomes, including late diagnosis of hip dysplasia and surgical intervention for hip dislocations [38]. Also, a large randomized controlled study in Norway on ultrasound screening showed that the prevalence of late subluxation/dislocation was 0.3 out of 1000 for universal ultrasound screening, 0.7 out of 1000 for selective ultrasound screening and 1.3 out of 1000 in the absence of ultrasound screening [39].

In our study, we found that children from rural regions of the Republic of Kazakhstan, indigenous Kazakh nationality, using the national cradle in their everyday life, as well as from regions with low average incomes, were significantly more likely to be exposed to late detection of hip dysplasia. In our study, we used publicly available population census data based on the 2009 National Population Census of the Republic of Kazakhstan as an income indicator, since we usually do not collect data on patients' incomes directly. One of the limitations of our study is the use of retrospective design, which limited our data, especially with the demographic situation at the time of the study.

However, effective screening largely depends on the competence of a primary care professional (neonatologists, doctors in polyclinics) in conducting a hip joint examination and compliance with the proposed algorithms. Numerous studies have noted that training in pediatric orthopedics is inappropriate for both medical schools and primary health care residency programs [40–43].

It is necessary to conduct advanced training courses on hip dysplasia for general practitioners and pediatricians in order to improve the early diagnosis of hip dysplasia, to introduce the latest international clinical recommendations for the diagnosis and treatment of hip dysplasia in children of the American Academy, and to develop protocols for physical examination in newborns in the UK [44]. Since we found a discrepancy in the provision of medical care to children of families from rural regions, which is due to the inaccessibility of specialized doctors (pediatric orthopedists) and the wider use of tight swaddling in national traditions. For this, additional research is needed to assess the connection between various factors affecting access to medical care and coverage of orthopedic examinations of the child population in order to introduce more effective programs to increase the availability of orthopedic care in rural regions of the Republic of Kazakhstan and to revise algorithms for physical examination and ultrasound screening programs in primary care institutions.

Hip dysplasia is a common pathology of infancy, and early detection and treatment will remain key factors in reducing both the cost of medical care throughout life and as a reduction in the duration and severity of disability of the patient.

## Conclusions

The outpatient and inpatient care provided to children with orthopedic pathology, in particular children with hip dysplasia, is of insufficient quality, and there are still a large number of cases of late detection of children with hip dysplasia, especially in such regions of the Republic of Kazakhstan as the Turkestan region, which is associated with low income indicators of the population, the inaccessibility of highly specialized orthopedic care in rural areas, as well as the widespread use of the national cradle "besik" in everyday life, especially for children of parents with indigenous Kazakh nationality.

Considering the above, more qualitative further studies are needed to facilitate the early diagnosis of children with hip dysplasia to determine the role of demographic and socio-economic factors in limiting access to pediatric orthopedic care for children with hip dysplasia, so that we can eventually improve the indicators of early detection of hip dysplasia in risk groups.

It should be noted that our findings can be integrated into international practice, especially in Central Asian countries, where there are also similar living conditions and problems in providing orthopedic care to children, and to explore possible areas of further work in improving the early detection of patients with hip dysplasia.

## Data Availability

The datasets used and/or analysed during the current study are available from the corresponding author on reasonable request.
